# Alternative Action Organizations: Social Solidarity or Political Advocacy?

**DOI:** 10.1177/0002764218768855

**Published:** 2018-04-13

**Authors:** Marco Giugni, Maria Grasso

**Affiliations:** 1 University of Geneva, Geneva, Switzerland; 2 University of Sheffield, Sheffield, UK

**Keywords:** alternative action organizations, politicization, raising awareness, lobbying, protesting

## Abstract

This article investigates the involvement of alternative action organizations in three forms of political advocacy in an attempt to gauge their degree of politicization. These forms can be understood as representing three different ways of making political claims: by raising public awareness with respect to a given cause or issue, by trying to influence the policy maker through “insider” lobbying activities, and by protesting in the streets as “outsiders.” Our findings show strong cross-national variations in all three forms of political activities, although not always following a consistent pattern. They also suggest that there is a relationship between the severity of the economic crisis and the form of advocacy. Most important, our analysis suggests that the politicization of alternative action organizations depends both on certain internal characteristics such as their degree of formalization and professionalization, as well as their thematic focus, and the scope of their activities, and on the broader context in terms of economic crisis, austerity policies, and political opportunities. As regards the latter, we find an impact especially on lobbying and protesting.

## Introduction

This article investigates the degree of politicization of alternative action organizations (AAOs) during the economic crisis in Europe. AAOs can be defined as collective bodies which organize collective events carrying out alternatives to dominant socioeconomic and cultural practices with visible beneficiaries and/or participants and claims on their economic and social well-being, including basic needs, health, and lifestyles. However, it is not clear to what extent they can be considered as political actors. On one hand, they engage in a wealth of activities aimed at providing services for specific populations in need. On the other hand, they often engage in politically oriented activities. In other words, AAOs have both a social solidarity dimension and a political action dimension.

Knowing the factors—at both the organizational and contextual levels—favoring the politicization of organizations that support socially and culturally alternative ways of practicing economic exchanges is important because it tells us much about the conditions under which civil society actors become political actors. It also helps bringing together the social and political dimensions of such initiatives and practices. Previous research has shown that making sense of the interrelation of micro–macro linkages is important for making sense of political action, particularly, in the context of the recent economic crisis (Grasso & Giugni, 2016). In this article, we develop on this previous research by turning to an emergent form of sociopolitical organizations to understand to what extent linkages between the organizational and the contextual levels can help explain their political involvement as well.

In order to study their politicization, we examine the extent to which a random sample of AAOs in nine countries engage in political activities and what explains such a political engagement. Based on theory, we include among the potential determinants both organization-related features, such as for example, their internal structures and resources, and aspects of the wider context. Among the latter, we are particularly interested in looking at the impact of certain features of the economic context and the political opportunity structures such as the severity of the crisis, austerity policies enacted by governments, and other features of the political–institutional system. Multilevel modeling allows us to ascertain the effect on politicization of predictors pertaining to both the organizations themselves and their broader context.

The focus of our study is on AAOs engaging in political advocacy, as opposed to other types of activities such as providing service to their beneficiaries or purely internal organizational activities (which often are in preparation of advocacy activities). In other words, we examine the extent to which AAOs that provide services get involved in political activities. In this regard, we distinguish between three ways of engaging in advocacy: raising public awareness with respect to a given cause or issue, engaging in “insider” lobbying tactics, or taking part in more contentious protest activities as “outsiders.” These are three distinct, though not mutually exclusive, ways in which organizations can be politically engaged. Each of them has its own logic and might be accounted for by different features of the organizations themselves as well as of their broader environment. The aims of this article are first, to assess the extent to which AAOs engage in these three types of political activities and how this varies across the nine countries included in our study, also depending on the severity of the economic crisis faced by those countries. Second, we aim to examine whether and to what extent these forms of advocacy are associated with certain characteristics of AAOs such as their degree of formalization and professionalization, their focus on economic rather than other goals, and their scope, as well as to how they are channeled through the broader context of the economic crisis, austerity policies, and political opportunity structures.

## The Political Mobilization of Social Solidarity

There has been a growing interest in recent years in what is variously called social economy (Laville, 2010), solidarity (or solidary) economy, social resilience (Hall & Lamont, 2013), or, more recently and particularly, with respect to the economic crisis, alternative forms of resilience (Kousis & Paschou, 2017). All these notions refer in some ways to alternative economic practices, located at the crossroad of the political and the social, initiated by citizen groups and networks (Kousis & Paschou, 2017). They include a wide variety of innovative activities and social relations such as solidary bartering (Fernández Mayo, 2009), local exchange trading schemes (Granger, Wringe, & Andrews, 2010), local and alternative currencies (North, 2007) ethical banks (Tischer, 2013), local market cooperatives (Phillips, 2012), cooperatives for the supply of social services such as in health and education (Costa, Andreaus, Carini, & Carpita, 2012), alternative forms of production (Corrado, 2010), critical consumption (Fonte, 2013), spontaneous actions of resistance and reclaim (Dalakoglou, 2012), and the reproduction of cultural knowledge via oral and artistic expression (Lamont, Welburn, & Fleming, 2013).

In general, this body of works tends to focus on the social and economic sides of these kinds of initiatives and organizations. In other words, they are mostly seen as social or economic actors, often with a solidary aim. In this way, their political dimension is often overlooked. We call them AAOs to denote that we believe this kind of actor to be also political and following recent works (Kousis, Bosi, & Cristancho, 2016). The action part, in particular, is meant to refer to their political side. In other words, these are not simply organizations engaged in *alternative* activities and forms of production—whether social or economic—but they also have a political *action* component. This is the key assumption which we would like to submit to empirical scrutiny below.

The political dimension of the kind of organizations considered here, of course, has not been totally neglected in previous research. Some scholarship stresses this aspect as well. Forno and Graziano (2014), for example, look at what they call “sustainable community movement organizations” following a social movement perspective to show how such new collective initiatives empower consumer and producer networks on a smaller scale. Similarly, Andretta and Guidi’s (2017) analysis of local alternative consumerism practices in Italy also have a political dimension, developing alternative processes through civic food networks and leading to radical forms of food democracy. More generally, Kousis and Paschou (2017) underline both the political and nonpolitical features of citizens’ collective responses to hard economic times which may take the form of AAOs.

Here, we examine two kinds of potential determinants of the political involvement of AAOs. The first set of factors consists in certain internal characteristics of organizations. To help us identifying such organizational-related factors we can draw from the social movement literature and, more specifically from resource mobilization theory (see Edwards & McCarthy, 2004, for a review). This research tradition has long inquired into the internal structuring of social movement organizations and the importance of factors such as the amount of resources and degree of organization for the movements’ emergence and mobilization (Jenkins, 1983; McCarthy & Zald, 1977). In this vein, Kriesi (1996) has pointed to four aspects to be considered in the analysis of organizations’ development: organizational growth and decline, internal structuring, external structuring, as well as goal orientations and action repertoires. Here, we examine the impact of four organizational aspects on the politicization of AAOs which relates to their internal structuring and partly on their goal orientation.

The first two aspects are intended to capture the degree of formalization and professionalization of AAOs. We examine, on one hand, whether they have a written constitution and, on the other hand, whether they have paid staff working for the organization. This latter aspect to some extent also reflects the size of the organization, as larger organizations tend to have paid staff while smaller ones are less likely to do so. Organizational size has long been considered by sociologists and political scientists as an important aspect to be addressed (see Clemens & Minkoff, 2004, for a review of work on the role of organizations in social movement research). We may therefore expect organizational size and, more generally, formalization as well as professionalization to be associated to politicization.

A third internal factor has to do with the goal orientation of AAOs. Here, we refer more specifically to the main thematic focus of their activities. There are obviously many different ways to classify an organization’s goals. For this study, we capture this dimension by distinguishing between economic activities and other activities. To be sure, this is a very rough distinction that does not do justice to the variety of aims and activities of AAOs. Yet given our focus on the economic crisis, it is a relevant one. Here, we may generally expect AAOs that focus on economic activities to be less politically oriented than other AAOs, other things being equal, as such a focus diverts their attention from political action.

The fourth and final internal factor we take into account in our analysis is the scope of activities of AAOs. Some organizations have a local scope, while others reach out to the regional, national, or sometimes even supranational level. We expect the scope of action to influence their degree of politicization. More specifically, we predict that local organizations will be less politicized than organizations that have a broader scope as they will be more focused on providing services than engaging in political advocacy. This should hold especially for raising awareness and lobbying, as these kinds of activities are more effective when there is a large public, respectively, when they target more powerful political elites, while protest activities can also be effective at the local level.

In addition to these organization-related factors, we are interested in examining the role of the larger context. AAOs do not act in a vacuum. Rather, their behavior is channeled through certain features of their broader environment. Therefore, we expect the extent of their political involvement to be influenced by such contextual features. Following previous research (Giugni & Grasso, 2016), here we focus in particular on three aspects of the context within which AAOs act. These three factors are aimed to capture, respectively, the severity of the economic crisis, austerity policies enacted by governments, and features of the political–institutional system.

The first two contextual factors which we expect to influence the politicization of AAOs refer to the severity of the crisis according to macroeconomic indicators. The economic crisis that started in 2008 has led to growing unemployment and shrinking economic growth across Europe and the rest of the world (De Grauwe & Ji, 2013). To get a grasp on this aspect we therefore rely on two standard indicators: gross domestic product (GDP) and the unemployment rate. These two indicators provide a measure of the severity of the crisis in terms of reduced economic growth, respectively, in terms of rising unemployment.

The involvement of AAOs in politically oriented activities such as raising awareness, lobbying, or protesting may also depend on certain features of the political–institutional context. Students of social movements have long shown the impact of this kind of factors pertaining to the broader environment of movements as providing political opportunities for mobilization (Eisinger, 1973; Kitschelt, 1986; Kriesi, Koopmans, Duyvendak, & Giugni, 1995; McAdam, 1996; Tarrow, 2011; see Kriesi, 2004, and Meyer, 2004, for reviews). Factors such as the relative openness or closure of the institutionalized political system, the stability or instability of elite alignments, the presence or absence of elite allies, and the state’s capacity and propensity for repression have most often been examined by scholars (McAdam, 1996). These aspects all refer to the input side of political opportunity structures. Additionally, albeit more rarely, some have pointed more specifically to the role of public policies as a key component of political opportunity structures capturing their output side (Meyer, 2004). Following the latter perspective, here we examine the impact of austerity policies as opening up or closing down opportunities for AAOs to get involved in political advocacy. We look more specifically at policies relating to social spending and taxation. Government expenses for social policies and the tax wedge may be seen as reflecting a definition of austerity policies as reducing government spending, especially in the social realm, and increasing taxation, especially on labor. They capture the output side of political opportunity structures.

While scholarship has often considered political opportunities as being “objective” aspects of the context that affect the mobilization of social movements, others have stressed their “subjective” side as well as perceived opportunities (Alimi, 2007; Banaszak, 1996; Kurzman, 1996, 2004; Lee, 2010; McAdam, 2004). Clearly, opportunities must be “framed” and perceived to have an impact on social movements (Gamson & Meyer, 1996). We therefore expect the politicization of AAOs to be associated to the perception of political opportunities. More specifically, we look at perceptions of the stability of the political system or the perceptions of the effectiveness of the government that play a role in this context. Again, the former may be considered as an input measure of perceived opportunities, while the latter capture their output side. Following social movement research, such perceptions of the openness or closedness of the political system in terms of government stability and effectiveness should have an impact especially on the propensity of AAOs to get involved in protest activities. However, we also expect them to influence their lobbying, as this is another, more institutionalized form of political intervention. Activities aimed to raising awareness, in contrast, should be less directly related to the perceptions of political opportunity structures.

## Data and Method

The data used in our analysis were retrieved in the context of the project “Living with Hard Times: How Citizens React to Economic Crises and Their Social and Political Consequences” (LIVEWHAT), funded by the European Commission under the auspices of the 7th Framework Programme. They consist of a sample of 4,297 AAOs whose characteristics such as organizational structure, aims, activities, and so forth were coded on information retrieved on their websites. AAOs were drawn from related national hubs/subhubs as identified by each national team and ranked according to two criteria: inclusiveness and diversity in terms of geographic origin and alternative action types coverage, along with the number of websites they contain. AAOs’ websites have been extracted from the databases of the highest ranked hubs/subhubs through a systematic process and the resulting national populations have been checked for their adequacy in terms of the above-mentioned criteria, with a preview of their geographic dispersion and the percentages each action type contains.

National random samples were generated from each country’s AAO websites. AAOs were coded only to the extent that they are active within the time frame of the recent global economic crisis (i.e., at least between 2007 and 2016) and offer related information as above. A structured protocol was used for the coding. It included information about the organizational profile, activities and beneficiaries, aims and solidarity orientations, and other related information (see LIVEWHAT, 2016, for more details).

Our dependent variables are meant to measure the politicization of AAOs through their engagement in three forms of advocacy. More precisely, we created three dummy variables based on information about the proposed routes to achieve their aims. The possible routes include the following: protest actions; direct actions; raising awareness; lobbying; policy reform, change, creation focusing on a number of specific issues; change government; change the establishment; and other routes. Here, we focus on three of them: raising awareness, lobbying, and protesting. Each of them reflects a distinct way to make political claims: respectively, by addressing the public opinion, by attempting to influence the political decision-makers, and by engaging in more contentious activities as outsiders.

We include the following independent variables in our analysis according to our discussion above. At the level of AAOs, we include four variables to measure their degree of formalization and professionalization through the presence of a written constitution, paid staff, their thematic focus by distinguishing between economic (to reduce the negative impacts of the economic crisis, austerity cuts; to reduce poverty and exclusion; to promote alternative economic practices, lifestyles and values, economic empowerment; and to promote alternative noneconomic practices, lifestyles, and values) and other (all remaining) activities, and the scope of their activities through an ordinal variable (local, regional, national, supranational). Information on these four variables stems from our content analysis of websites of AAOs. At the contextual level, we include six variables: the quarterly GDP as percentage change from the previous period and the unemployment rate as percentage of the labor force to capture the economic crisis, the public social spending as percentage of the GDP and the tax wedge as percentage of the labor cost to grasp austerity policies, and the political stability index and the government effectiveness index to measure perceived political opportunities. Information on these six variables, which varies across countries, was retrieved on https://data.oecd.org and http://www.theglobaleconomy.com. The contextual measures refer to 2014.

Our analysis is divided in two parts: a first, descriptive part in which we show variations in the three forms of advocacy across countries, also in relation to the severity of the economic crisis in those countries, and a second, more explanatory part in which we run a number of multilevel logistic regression models for each of the three types of political activities. The country forms the group variable in the multilevel regressions. We include in the analysis the following countries: France, Germany, Greece, Italy, Poland, Spain, Sweden, Switzerland, and the United Kingdom. These countries were hit to a different extent by the economic crisis (Giugni & Grasso 2018). In particular, the three southern European countries were much more harshly hit, while the other countries had to face a weak to intermediate crisis. Furthermore, the same countries that were more strongly hit by the crisis are also those in which austerity policies implemented by governments have gone the farthest.

## Three Types of Political Activities of Alternative Action Organizations and Their Variations Across Countries

This section examines variations in the use of the three forms of advocacy by AAOs across countries as well as across degrees of severity of the economic crisis. In addition, we also show how the characteristics of AAOs vary according to these two criteria.

Table 1 shows how the three types of political activities vary across the nine countries of our study. Overall, we observe a much higher frequency in raising awareness in all nine countries, while both lobbying and protest activities are being used less often. Most important, there are important differences across countries in all three forms. Raising awareness is more frequent in Poland, followed by Greece and Spain. Swedish and Swiss AAOs also make a fair usage of this form of advocacy, whereas the latter is less frequent in France, Germany, and Italy.

**Table 1.** Cross-National Variations of Three Types of Political Activities of Alternative Action Organizations (Percentages).

	France	Germany	Greece	Italy	Poland	Spain	Sweden	Switzerland	United Kingdom
Raising awareness	42.8	37.6	64.6	42.2	75.1	61.0	50.5	55.6	41.2
Lobbying	2.2	8.6	3.00	1.8	4.6	2.8	18.9	12.6	15.2
Protesting	2.2	4.2	28.80	19.6	9.8	32.5	3.1	9.0	2.0
*N*	500	498	500	500	498	459	509	333	500

*Note*. Pearson χ^2^ = 256.563, Pr = .000, Cramer’s *V* = 0.244 (raising awareness). Pearson χ^2^ = 225.736, Pr = .000, Cramer’s *V* = 0.229 (lobbying). Pearson χ^2^ = 496.614, Pr = .000, Cramer’s *V* = 0.340 (protesting).

Similarly important and perhaps even stronger variations exist concerning lobbying. Here, we observe a strong contrast between Sweden, Switzerland, and United Kingdom, on one hand, and all other countries (with the partial exception of Germany), on the other. While AAOs in the former three countries are strongly committed to lobbying activities, this form of advocacy is less popular in the other countries.

The cross-national variations in the use of protest activities are perhaps even more striking. Moreover, here we can observe a clear-cut pattern: AAOs in the three southern European countries seem to be much more protest-oriented than elsewhere. Thus, Italian, but especially Greek and Spanish AAOs make use of protest activities to a much larger extent than their counterparts in the other six countries. This suggests that these similarities and differences group in some way according to the severity of the crisis faced by the countries, at least for some of these political activities. This holds in particular for lobbying and protesting, as lobbying activities are most often used when the crisis was weak, while protest activities are much more frequent in countries facing a strong crisis.

Before we move to the regression models, we would like to take a look at variations in the organizational variables included in the models. Table 2 shows how the two indicators of the degree of formalization and professionalization of AAOs as well as their focus on economic aims vary across the nine countries. All three variables show strong cross-national variations, suggesting that context impacts on specific features of AAOs. Greek, but especially Spanish and British AAOs are characterized by a lower level of formalization as not many of them possess a written constitution. In the case of the United Kingdom, this might be explained by the constitutional tradition of the countries (the United Kingdom does not have a written constitution), while the other two countries might suggest a link with the economic crisis (but see the case of Italy).

**Table 2.** Cross-National Variations in Three Characteristics of Alternative Action Organizations (Percentages).

	France	Germany	Greece	Italy	Poland	Spain	Sweden	Switzerland	United Kingdom
Written constitution	28.2	24.3	10.4	36.8	27.3	4.8	36.4	39.0	2.8
Paid staff	13.4	8.4	7.2	5.8	12.5	4.6	52.5	36.6	30.8
Economic aim	78.0	76.7	57.4	76.8	57.8	72.6	50.9	75.1	50.0
*N*	500	498	500	500	498	459	509	333	500

*Note*. Pearson χ^2^ = 717.543, Pr = .000, Cramer’s *V* = 0.409 (paid staff). Pearson χ^2^ = 413.552, Pr = .000, Cramer’s *V* = 0.310 (written constitution). Pearson χ^2^ = 243.449, Pr = .000, Cramer’s *V* = 0.238 (economic aim).

These differences are only partly reflected in the presence of paid staff. Here, we observe clearly a higher share of AAOs that have paid staff in Sweden, Switzerland, and the United Kingdom, while this share is much smaller in all other countries and especially so in Germany, Greece, Italy, and Spain. In other words, AAOs in these countries seem less professionalized than in the other countries.

Overall, most of the AAOs tend to focus on economic aims rather than on other aims such as, for example, combating discrimination, increasing tolerance and mutual understanding, or promoting alternative noneconomic practices, lifestyles, and values. In particular, Greek AAOs (but also Polish, Swedish, and British ones) are less focused on economic aims than their counterparts in other countries. These organizational characteristics could be associated to the severity of the economic crisis. However, this might be true of the two indicators of formalization and professionalization, but not for the focus on economic aims. Yet the two indicators point to opposing directions. While AAOs in countries more strongly affected by the crisis were more likely to have a written constitution than their counterparts in more weakly affected countries, they also are less likely to have paid staff than in the other two situations.

Finally, we can take a look at the scope of the activities carried out by AAOs. Table 3 shows the cross-national variations of this variable. Overall, most organizations focus on the local level. This is hardly surprising as this is the level where the kind of activities carried out by AAOs are most effective. Activities at the regional and national levels vary depending on the country, while supranational activities are less frequent than those referring to the other three levels except in one country, namely Switzerland.

**Table 3.** Cross-National Variations of the Scope of Activities of Alternative Action Organizations (Percentages).

	France	Germany	Greece	Italy	Poland	Spain	Sweden	Switzerland	United Kingdom
Local	69.1	81.1	79.5	84.4	57.2	73.9	61.0	67.3	61.5
Regional	7.9	7.6	13.1	5.1	30.3	19.2	17.6	19.0	10.7
National	17.4	10.0	6.2	7.6	9.2	5.0	20.4	4.3	24.8
Supranational	5.6	1.3	1.2	3.0	3.2	1.9	1.0	9.3	3.1
*N*	482	450	482	436	435	422	490	300	488

*Note*. Pearson χ^2^ = 430.809, Pr = .000, Cramer’s *V* = 0.190.

In terms of cross-national variations, we observe a higher share of local activities especially in Germany, Greece, and Italy. However, differences are not huge. They are more substantial when it comes to the regional and national levels. Regional activities are particularly important in Poland, but to some extent also in Spain, Sweden, and Switzerland, while national activities are more frequent in France, Sweden, and the United Kingdom. Finally, as already noted, Switzerland stands out as regards supranational activities, followed by France, while this level is less important in the other countries. This suggests that the scope of activities of AAOs might be in part related to the severity of the economic crisis. Specifically, local activities are more frequent in countries more strongly affected by the crisis. Regional and national activities, in contrast, seem more popular where the crisis was not as strong, but the patterns are far from being clear-cut in this regard. Supranational activities are also less frequent in countries that have faced a stronger crisis, although their overall level is lower in all countries except in Switzerland.

## Explaining the Political Orientation of Alternative Action Organizations

The main goal of this article is to gauge the potential impact of certain characteristics of AAOs, and particularly, the role of certain contextual features for explaining whether AAOs engage in the three different forms of political advocacy. We do so by means of three sets of random-intercept logistic regressions whereby observations are clustered by country. Each set is made of seven models: The first one only includes the organizational predictors, while each of the six subsequent models adds in turn one of the six contextual predictors. The latter are included one by one in separate models to avoid possible multicollinearity problems. The coefficients shown are odds ratios, which lend themselves to interpretation more easily than log-odds (when the odds ratio is greater than 1 the effect is positive, when it is smaller than 1 it is negative, and when it equals 1 there is no difference).

Table 4 shows the results for raising awareness as dependent variable. As we can see in the first model, most of the organizational variables have a significant effect on this form of advocacy. A certain degree of formalization, as seen in the existence of a written constitution, seem to favor the engagement of AAOs in activities geared toward raising awareness toward their aims. Having paid staff, however, has no significant effect, suggesting that a higher degree of professionalization in this respect does not matter for these types of political activities. AAOs that have economic aims are significantly less likely to be committed to raising awareness than AAOs that privilege other aims. Finally, locally oriented AAOs are significantly less likely to engage in these types of activities than those whose activities have a broader scope. This can be seen in the significant and positive coefficients for regional, national, and supranational scope as opposed to the local scope, which is the reference category of this variable. All these effects hold across the other models.

**Table 4.** Multilevel Logistic Regression Models Explaining Raising Awareness (Odds Ratios).

	Model 1	Model 2	Model 3	Model 4	Model 5	Model 6	Model 7
*Organizational level*							
Written constitution	1.839*** (0.159)	1.839*** (0.159)	1.845*** (0.160)	1.841*** (0.160)	1.840*** (0.159)	1.841*** (0.160)	1.838*** (0.159)
Paid staff	1.140 (0.110)	1.140 (0.110)	1.146 (0.110)	1.135 (0.109)	1.135 (0.109)	1.142 (0.110)	1.147 (0.110)
Economic aims	0.715*** (0.053)	0.715*** (0.053)	0.714*** (0.053)	0.716*** (0.053)	0.715*** (0.053)	0.715*** (0.053)	0.715*** (0.053)
Scope (ref.: local)							
Regional	1.441*** (0.146)	1.441*** (0.146)	1.447*** (0.147)	1.440*** (0.146)	1.440*** (0.146)	1.443*** (0.146)	1.445*** (0.146)
National	2.321*** (0.257)	2.321*** (0.257)	2.323*** (0.257)	2.320*** (0.257)	2.320*** (0.257)	2.320*** (0.257)	2.321*** (0.257)
Supranational	1.874** (0.376)	1.874** (0.376)	1.883** (0.377)	1.870** (0.375)	1.869** (0.375)	1.876** (0.376)	1.880** (0.377)
*Contextual level*							
GDP		1.000 (0.130)					
Unemployment			1.044* (0.0213)				
Social spending				0.936 (0.0414)			
Tax wedge					0.979 (0.0211)		
Political stability						0.763 (0.379)	
Government effectiveness							0.649 (0.190)
							
Constant	0.985 (0.198)	0.985 (0.252)	0.591* (0.175)	5.248 (5.927)	2.330 (2.050)	1.169 (0.435)	1.702 (0.701)
Sigma_u	0.566	0.566	0.459	0.504	0.536	0.557	0.505
Rho	0.089	0.089	0.060	0.072	0.080	0.086	0.072
Log likelihood	−2557.961	−2557.961	2556.173	−2556.956	−2557.485	−2557.815	−2556.981
Observations	3,985	3,985	3,985	3,985	3,985	3,985	3,985
No. of countries	9	9	9	9	9	9	9

* *p* < .05. **p < .01. ***p < .001.

When we introduce the six contextual variables in the other models, the results are less clear-cut. Of the six contextual predictors, only one displays a statistically significant effect, namely unemployment, although the magnitude of the effect is not very large. Thus, raising awareness, as a form of advocacy, does not seem to be very much influenced by the broader context, neither in terms of economic crisis, nor in terms of austerity policies or political opportunities.

Table 5 shows the results for lobbying. In this case, all organizational predictors have a statistically significant effect, including the presence of paid staff, which makes the engagement of AAOs in this type of political activities more likely. The effect of the other variables is similar to the one observed earlier. Here we find however more effects on the side of the contextual predictors as GDP growth and the two measures of political opportunities (political stability and government effectiveness) are all significantly and positively associated with a higher likelihood to engage in lobbying. Concerning the latter two, in particular, the higher the perceived political stability and government effectiveness, the more likely are AAOs to engage in lobbying activities. In addition, at a 90% significance level, the unemployment rate, social spending, and tax wedge also have an impact. Thus, if we relax the criteria for statistical significance, all contextual variables seem to matter for this type of political activity.

**Table 5.** Multilevel Logistic Regression Models Explaining Lobbying (Odds Ratios).

	Model 1	Model 2	Model 3	Model 4	Model 5	Model 6	Model 7
*Organizational level*							
Written constitution	2.366*** (0.359)	2.349*** (0.355)	2.328*** (0.353)	2.368*** (0.359)	2.370*** (0.359)	2.304*** (0.351)	2.327*** (0.351)
Paid staff	1.557** (0.230)	1.554** (0.228)	1.553** (0.228)	1.565** (0.231)	1.553** (0.229)	1.551** (0.228)	1.543** (0.226)
Economic aims	0.667** (0.089)	0.672** (0.090)	0.665** (0.090)	0.667** (0.089)	0.666** (0.089)	0.663** (0.089)	0.656** (0.088)
Scope (ref.: local)							
Regional	3.280*** (0.562)	3.254*** (0.558)	3.292*** (0.565)	3.253*** (0.558)	3.256*** (0.558)	3.267*** (0.560)	3.290*** (0.564)
National	5.316*** (0.853)	5.287*** (0.847)	5.295*** (0.849)	5.344*** (0.857)	5.335*** (0.856)	5.348*** (0.858)	5.298*** (0.849)
Supranational	4.967*** (1.411)	4.936*** (1.400)	4.893*** (1.389)	4.927*** (1.401)	4.886*** (1.390)	4.900*** (1.391)	4.812*** (1.367)
*Contextual level*							
GDP		1.448** (0.204)					
Unemployment			0.946 (0.0285)				
Social spending				0.901 (0.0553)			
Tax wedge					0.954 (0.0265)		
Political stability						3.260* (1.919)	
Government effectiveness							2.825*** (0.869)
Constant	0.028*** (0.00846)	0.018*** (0.00545)	0.055*** (0.0239)	0.394 (0.615)	0.187 (0.211)	0.0135*** (0.00619)	0.008*** (0.00354)
Sigma_u	0.775	0.559	0.640	0.670	0.668	0.629	0.475
Rho	0.154	0.087	0.111	0.120	0.120	0.107	0.064
Log likelihood	−880.352	−877.748	−878.951	−879.055	−879.061	−878.675	−876.662
Observations	3,985	3,985	3,985	3,985	3,985	3,985	3,985
No. of countries	9	9	9	9	9	9	9

* *p* < .05. **p < .01. ***p < .001.

Finally, Table 6 shows the result for protesting. In this case, the organizational predictors have a lower explanatory power as only the aims and the scope of activities are statistically significant. Specifically, just as for raising awareness and lobbying, AAOs who have economic aims are less likely to be engaged in protesting than AAOs who have other aims. Moreover, AAOs with regional activities are also less likely to do this form of advocacy than those with a local scope. Concerning the contextual predictors, three of them are significantly associated with protesting. GDP growth and perceived government effectiveness make protesting less likely, while unemployment makes it more likely. It is interesting to note that the latter variable has an opposite effect than when used to explain lobbying.

**Table 6.** Multilevel Logistic Regression Models Explaining Protesting (Odds Ratios).

	Model 1	Model 2	Model 3	Model 4	Model 5	Model 6	Model 7
*Organizational level*							
Written constitution	0.877 (0.129)	0.878 (0.129)	0.890 (0.131)	0.878 (0.129)	0.878 (0.129)	0.881 (0.129)	0.878 (0.129)
Paid staff	0.687 (0.137)	0.691 (0.138)	0.690 (0.137)	0.686 (0.137)	0.686* (0.137)	0.690 (0.138)	0.696 (0.139)
Economic aims	0.640*** (0.071)	0.637*** (0.071)	0.642*** (0.072)	0.641*** (0.071)	0.640*** (0.071)	0.641*** (0.071)	0.642*** (0.072)
Scope (ref.: local)							
Regional	0.390*** (0.0727)	0.392*** (0.0730)	0.390*** (0.0728)	0.390*** (0.0726)	0.390*** (0.0727)	0.390*** (0.0728)	0.391*** (0.0728)
National	1.003 (0.193)	1.001 (0.193)	0.994 (0.191)	1.004 (0.193)	1.003 (0.193)	1.000 (0.193)	0.999 (0.192)
Supranational	0.608 (0.224)	0.609 (0.225)	0.615 (0.227)	0.608 (0.224)	0.608 (0.224)	0.610 (0.225)	0.612 (0.226)
*Contextual level*							
GDP		0.627* (0.139)					
Unemployment			1.123*** (0.0375)				
Social spending				0.957 (0.0984)			
Tax wedge					0.993 (0.0471)		
Political stability						0.342 (0.337)	
Government effectiveness							0.290* (0.156)
Constant	0.142*** (0.0584)	0.252*** (0.108)	0.036*** (0.0177)	0.429 (1.125)	0.190 (0.368)	0.281 (0.207)	0.679 (0.507)
Sigma_u	1.185	0.958	0.752	1.174	1.184	1.107	0.930
Rho	0.299	0.218	0.147	0.295	0.299	0.271	0.208
Log likelihood	−1232.673	−1230.869	−1228.894	−1232.583	−1232.661	−1232.122	−1230.582
Observations	3,985	3,985	3,985	3,985	3,985	3,985	3,985
No. of countries	9	9	9	9	9	9	9

* *p* < .05. **p < .01. ***p < .001.

## Conclusion

This article has investigated the involvement of AAOs in three forms of political advocacy in an attempt to gauge their degree of politicization. These forms can be understood as representing three different ways of making political claims: by raising public awareness with respect to a given cause or issue, by trying to influence the policy maker through “insider” lobbying activities, and by protesting in the streets as “outsiders.” We first conducted descriptive analyses showing how AAOs’ engagement in raising awareness, lobbying, and protesting vary across countries, also in relation to the severity of the economic crisis in those countries. Second, we performed a number of multilevel logistic regression models for each of the three types of political activities.

Our findings show strong cross-national variations in all three forms of political activities, although not always following a consistent pattern. They also suggest that there is a relationship between the severity of the economic crisis and the form of advocacy. In particular, lobbying is more frequent in those countries less severely hit by the crisis, whereas protesting is more often used in countries that were more harshly hit. We also showed variations in certain characteristics of AAOs such as their degree of formalization and professionalization, or their focus on economic aims, and the geographical scope of their activities across countries and depending on the severity of the economic crisis. In particular, possessing a written constitution was more frequent in countries that experienced a deeper crisis, whereas having paid staff was more often observed in countries that faced a weaker crisis.

Most important, our analysis suggests that the politicization of AAOs depends both on certain internal characteristics such as their degree of formalization and professionalization, as well as their thematic focus, and the scope of their activities, and on the broader context in terms of economic crisis, austerity policies, and political opportunities. As regards the latter, we found an impact especially on lobbying and protesting. To be sure, there are many other factors—both internal and external—that may determine or at least channel the politicization of AAOs, as that of any other kind of organization for that matter. For example, on the internal side, this might depend on the specific and often contingent composition of the organizations’ directorates, who might be more or less inclined to invest in political activities. Also, there might be path dependency insofar as the kinds of activities carried out by a given organization at time *t*_1_ might be influenced by whether they used to do previously, at time *t*_0_. On the external side, the national political culture as well as other aspects of the political opportunity structure, such as, for example, the configuration of political alignments at a given moment in time (Kriesi et al., 1995; Tarrow, 1989) might either encourage or discourage AAOs to get involved in political activities. In this article, however, we were mostly interested in examining the connections between the degree of internal structuration, thematic focus and the action scope of AAOs, their economic and institutional context, and the likelihood that they become active on the political scene. This stemmed from a theoretical choice but was also partly constrained by the availability of information in our data.

In addition to the limitations stemming from the information available in the data, we should also stress that our analysis rests on the way in which AAOs have been sampled. While we cannot say to what extent and how, for sure sampling affects our findings. For example, by including only organizations that have online presence, our sample might leave out certain types of other organizations. Furthermore, it was hard to determine to what extent the organizations included in the sample have a long tradition of contentious political action, as opposed to others that are newcomers in this respect. We nonetheless believe that we have provided evidence of the political engagement, of AAOs, in addition to their social engagement, and how their degree of politicization varies depending on the types of political activities as well as depending on certain of their organizational features and of their broader environment.
